# Ways to improve treatment effectiveness of post-stress disorders, that developed as a result of hostilities, using transcranial magnetic stimulation of the brain

**DOI:** 10.1192/j.eurpsy.2026.11880

**Published:** 2026-07-31

**Authors:** V. Kuzminov, I. Linskiy, V. Zadorozhny, R. Lakinskiy, M. Denysenko, T. Tkachenko, O. Minko, V. Borzenko

**Affiliations:** 1 Department of emergency psychiatry and narcology; 2 Department of emergency psychiatry and narcology, SI “Institute of Neurology, Psychiatry and Narcology named after P.V. Voloshin of the NAMS of Ukraine”, Kharkiv, Ukraine

## Abstract

**Introduction:**

Under the influence of powerful psych traumas, which always accompany combat operations, there are numerous comorbid combinations of post-stress disorders with different psychopathology (anxiety, depressive states, obsessions, insomnia, nightmares etc.). The low effectiveness of treatment of these disorders forces the development of new approaches in the treatment of these disorders.

**Objectives:**

Transcranial magnetic stimulation of the brain were used in complex therapy of post-stress disorders in combatants (42) and civilians (45) who suffered from Russian aggression. Virtual reality methods are being developed for the treatment of this group of patients.

**Methods:**

Psychopathological, pathopsychological assessment methods

**Results:**

Transcranial magnetic stimulation was used in combination with standard therapy (pharmacotherapy and psychotherapy). A more rapid reduction, primarily of depressive syndrome, was noted compared to the control group. The reduction in anxiety was not statistically significant across study groups. No change in sleep duration was noted. However, patients who received Transcranial magnetic stimulation in complex therapy reported satisfaction with sleep and a reduction of nightmares. Good adherence to therapy was observed in patients receiving Transcranial magnetic stimulation.

**Conclusions:**

Encouraging data were obtained regarding the optimization of treatment of post-stress disorders using transcranial magnetic stimulation of the brain.It is assumed that the implementation of new treatment methods such as: virtual reality, visual-perceptual subsensory psychocorrective influence and others will be effective in treatment PTSD.

**Disclosure of Interest:**

None Declared

